# Improving the Depth and Reliability of Glycopeptide Identification Using Protein Prospector

**DOI:** 10.1016/j.mcpro.2025.100903

**Published:** 2025-01-07

**Authors:** Robert J. Chalkley, Peter R. Baker

**Affiliations:** Department of Pharmaceutical Chemistry, University of California, San Francisco, USA

**Keywords:** glycopeptides, glycoproteomics, glycopeptide ID, database searching, Protein Prospector

## Abstract

Glycosylation is the most common and diverse modification of proteins. It can affect protein function and stability and is associated with many diseases. While proteomic methods to study most post-translational modifications are now quite mature, glycopeptide analysis is still a challenge, particularly from the aspect of data analysis. Several software packages have been developed in the last few years that aim to support omic-level N-linked glycopeptide analysis. This study presents developments of Protein Prospector for the analysis of N-glycopeptide data. Results are compared to other software, showing that Protein Prospector reports many more glycoforms of glycopeptides than competing software. The advantages and disadvantages of considering glycan adducts are also evaluated, showing how considering them can correct previously wrong assignments.

## Highlights

• New glycopeptide analysis pipeline in Protein Prospector.
• Protein Prospector identifies more glycoforms per glycosylation site.
• Large number of glycopeptide adduct spectra were identified.
• Adduct spectra are misinterpreted as different glycan by some software.

## Graphical Abstract

**Figure:**
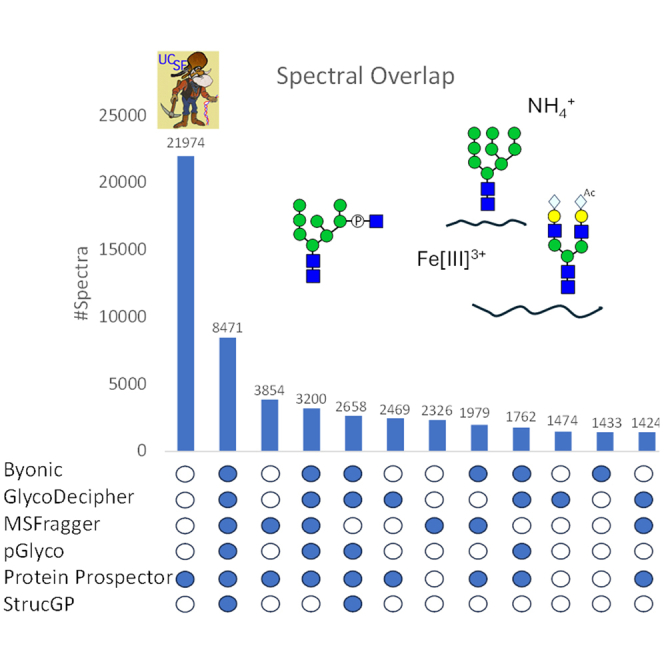

## In Brief

A new glycopeptide analysis pipeline is presented for the Protein Prospector software, and its performance is compared to other tools. It is shown to identify more glycopeptides, mostly through identifying additional glycoforms of peptides also found by other software. Many adduct spectra are identified and evidence is presented that some software misinterprets these spectra as other glycans if they do not consider the adduct as a possibility.

Glycosylation of proteins is the most common post-translational modification the cells use to regulate their function, activity, and stability. Inside the cell the addition of a single N-acetylglucosamine (GlcNAc) to nuclear, cytoplasmic and mitochondrial proteins is a common regulatory modification and is associated with diseases ranging from diabetes to Alzheimers (1). Extended sugar structures are added to extracellular proteins. These are mostly either O-linked (to serine, threonine or tyrosine) or N-linked through asparagines. They can be quite diverse and large (particularly in the case of glycosaminoglycans). The modifications can affect protein folding and trafficking, as well as protein interactions, and are dysregulated in cancer, autoimmune and neuropathological disorders (2).

In mammals, N-linked glycosylation is easier to analyze than O-linked for three main reasons: (1) It is added in a consensus motif of N-X-S/T, where X is any amino acid other than a proline, so sites of modification can be predicted. In contrast, O-glycosylation sites cannot be projected based on sequence, and it is common to have multiple modification sites in close proximity, (2) The same initial sugar structure is always added in N-glycosylation, before being trimmed down in the ER and then optionally further modified in the golgi, meaning a common core of five sugar residues is known and many sugar structures can be predicted based on glycan residue composition, and (3) The glycosidic link between the asparagine and first GlcNAc in the core is relatively stable so typically stays intact during N-glycopeptide fragmentation in a mass spectrometer, whereas the O-glycosidic link is less stable than the peptide backbone, so in collisional-based fragmentation is broken before peptide fragmentation, meaning evidence of site/s of modification is lost unless radical-based fragmentation methods such as ETD are used (3).

Protein Prospector is a set of tools including a MSMS database search engine (Batch-Tag) which has been used for peptide analysis for the last 30 years (4). It was one of the first software packages that supported glycopeptide analysis from complex mixtures (5). These studies made use of ETciD data where efficient peptide fragmentation is achieved but limited glycan fragmentation such that glycan assignment was mostly by mass alone. With the advent of stepped collision energy acquisition methods, it is now possible to get good fragmentation of both peptide and glycan in the same spectrum (3), so one can better evaluate the glycan component of the assignment while also achieving peptide backbone fragmentation.

There are many other glycopeptide analysis software packages available, and a community study was performed several years ago that evaluated the relative performance of some of these (6). In this study Protein Prospector was found to be a top performer for both N- and O-glycopeptide analysis, and Byonic also performed relatively well for N-glycopeptides. These two pieces of software are tools originally developed for analyzing unmodified peptides and those with simple modifications and were adapted for glycopeptide analysis. They first try to identify the peptide portion of a glycopeptide from peptide backbone fragment ions after allowing for precursor mass shifts consistent with any glycan in a considered list. They then try to explain additional unassigned fragment ions in the spectrum as being derived from glycan fragmentation.

Since this study, several other software packages have been developed (7, 8, 9, 10). Of note, some of these new software tools employ a different strategy that is more targeted toward N-glycopeptide analysis. As all larger N-glycopeptides start with two GlcNAc then three mannose attached to the modified Asn, and as glycans fragment readily upon collisional dissociation, a diagnostic pattern of fragment ions spaced by 203.08 Da and then 162.05 Da can be observed. Searching spectra for this pattern one can identify probable N-glycopeptide spectra and can infer the mass of the peptide component. This allows the software to search for a restricted set of potential peptides based on the mass and presence of an N-glycosylation modification motif, allowing the identification of glycopeptide spectra with limited peptide fragmentation information. The first of these ‘glycan-first’ software packages was pGlyco; GlycoDecipher, and StrucGP also follow this analysis approach.

For the most part, glycopeptide mass spectrometry fragmentation data assigns glycan compositions (number of N-acetylhexosamine (HexNAc); hexoses (Hex); deoxyhexose (dHex), (in the case of mammalian glycans this is always fucose (Fuc)); sialic acids such as N-acetylneuraminic acid (NeuAc) and N-glycolylneuraminic acid (NeuGc), plus other potential modifications on the glycan such as phosphorylation, acetylation or sulfation. In some cases, topological information can also be inferred (StrucGP (10) in particular tries to extract this information). The list of glycans considered is an important parameter in glycopeptide searching. Although some researchers acquire matched glycomic data to define glycans that should be considered (11), most use the default, generally larger, glycan databases offered by the software.

In this article, we present an improved glycopeptide analysis approach in Protein Prospector that allows the identification of more N-glycopeptide spectra from collisional fragmentation data. To evaluate its performance, a published dataset from mouse liver that has previously been analyzed by other glycopeptide software packages was reanalyzed. This enabled the results from six pieces of software to be compared: Byonic; GlycoDecipher; MSFragger; pGlyco3; Protein Prospector; and StrucGP. The results show that glycan search space is a significant parameter in identification success. The effect of considering salt/metal adducts to glycans when searching is also investigated.

## Experimental Procedures

### Experimental Design and Statistical Rationale

This is a reanalysis of previously published raw data for evaluating software performance. No biological conclusions are drawn from the results.

### Data

The data used in this publication were created for a previous publication (12) and shared *via* ProteomeXchange (13) as dataset PXD005553. Briefly, glycopeptides were enriched from 5 mouse liver samples using ZIC-HILIC, and then each was analyzed by a 6-h run acquired on a Fusion Lumos (Thermo) mass spectrometer using stepped HCD fragmentation (Collision Energy 30% ± 10).

### Protein Prospector Glycopeptide Searching and Identification

The glycopeptide identification workflow in Protein Prospector makes use of three modules within the software package: MS-Filter, Batch-Tag, and Search Compare. Batch-Tag is the main database search engine that is used for the identification of MSMS spectra of peptides. Search Compare is the software that is used to report these results and is where reporting thresholds such as peptide and protein FDR are specified, along with what columns/information is desired in the report. MS-Filter is software that can be used for filtering peak list files based on charge, presence of specific peaks, patterns of peaks, or mass losses from a precursor. It has previously been used in publications for filtering spectra for the presence of glycan oxonium ions and glycopeptide Y ions (14), and filtering for HexNAc oxonium ions is still typically the first step in a glycopeptide identification workflow. Peak lists from the raw data were created using the in-house software ‘PAVA’, which incorporates Monocle (15) for improved monoisotopic peak assignment. After creating a project for Batch-Tag analysis the user can run MS-Filter to create a subset index file that tells Batch-Tag which spectra contains peaks of interest and therefore should be searched. For most glycopeptide datasets, including the data analyzed in this publication, it is sufficient to filter for the presence of the HexNAc oxonium ion at m/z 204.087, where we allowed a 20 ppm mass tolerance. Users could alternatively or additionally require the presence of other HexNAc-related fragment ions such as m/z 138.055, which become more prominent if very high collision energies were used.

The identified set of potential glycopeptide spectra are then searched in Batch-Tag for reliable identification of glycosylated peptides. Protein Prospector has multiple glycan sets that can be selected for consideration. The names of these sets include separation by fragmentation type: in sets for ExD the software will assume glycans are retained on fragment ions, whereas in CID sets it assumes O-glycans will be completely missing from fragment ions, whereas N-glycans will retain the first core HexNAc attached to the Asn. They are also separated as Human or Mammals: the former does not consider NeuGc-containing glycans, whereas the latter does. On a local installation of Protein Prospector it is possible to add user-defined glycan sets to search. There are also options to add cations, which if selected will consider these for all glycans in the set. For the mouse liver dataset in this study the “N-glycosylation CID Mammals” set of glycans (730 entries, listed in Supplemental Table S1) was considered only on Asn in the glycosylation motif N[ˆP][ST]; *i.e.* the following residue could not be a proline, then either serine or a threonine. This set includes a large number of glycans, including those containing NeuGc, NeuGcAc (acetyl-N-glycolylneuraminic acid), NeuAc, NeuAcAc and phosphate, as well as permutations of HexNAc, Hex, and Fuc. The list of glycans in each set can be viewed in an expanded part of the submission form for both Batch-Tag and MS-Filter. For the Batch-Tag search, no cation adducts were considered. Search parameters, where practical, were matched to those used in a previous publication analyzing this data (9). Data was searched with a 5 ppm tolerance on the precursor ions and 20 ppm tolerance for fragment ions. All Mus Musculus entries in a SwissProt database downloaded on May 26th 2022 were considered, along with sequence-randomized decoy versions to allow FDR estimation, leading to 17,126 target and an equal number of decoy entries being considered. Peptides were required to be fully tryptic, allowing for up to 3 missed cleavages. Additional variable modifications considered (in addition to the glycosylation) included Oxidation (M); Gln-> pyro-Glu (N-term Q) and permutations of protein N-terminal methionine removal and/or acetylation. The “Glycopeptide Peak Filtering” option was selected: this tells the software to filter out glycan oxonium ions when doing the peptide identification. This search essentially only evaluates the peptide fragmentation although it does also score the presence of the Y1 ion for a N-glycopeptide (or the Y0 for an O-glycopeptide). The glycan assignment at this stage is based on mass alone.

Search results were thresholded to a 1% FDR threshold at both the protein and the unique peptide level based on target:decoy search results using Search Compare. Within Search Compare the ‘MS-Filter Y0 list’ option under Glycosylation parameters was selected. This creates a link to an MS-Filter submission in the Search Compare peptide report and populates it with the list of peptide sequences of glycopeptides identified in these search results. In addition to the peptide sequence, it also includes any identified variable modifications other than glycans, the protein accession/s the peptide is derived from, the number of peptides identified to the protein, the mean retention time of identified glycoforms of this peptide and the time spread of different glycoforms identified. This allows the user to run an MS-Filter analysis looking for Y0/Y1/Y2 fragments (i.e. intact peptides with 0–2 core glycans retained) for all glycopeptides identified. For the present analysis only Y1 was required within a 20 ppm tolerance, a minimum MS-Tag score threshold of 0 and Glyco score of 5. The same ‘N-glycosylation CID mammals’ glycan list as used in the Batch-Tag search was considered, but in addition ammonium (+Cation:N(1)H(4)); iron (+Cation:Fe[III]) and aluminum (+Cation:Al[III]) adducts were permitted, as well as allowing for a precursor offset of ± 1; *i.e.* allowing for the monoisotopic peak to be incorrect by plus or minus an isotope peak.

This MS-Filter search goes through the peak list files (filtered for presence of an m/z 204 peak) and finds spectra that contains a peak at the expected mass of the Y1 peak for a glycopeptide confidently identified in the Batch-Tag search. It then scores this result for peptide fragments and glycan fragments. The glycan scoring is described in Supplemental File S1. Briefly, the scoring is based on how many B and Y ions are consistent/inconsistent with a particular glycan assignment. In the case of sialic acid oxonium ions, as these are so common as background, observation of these in an assignment not containing a sialic acid is not penalized, whereas lack of these in a sialylated assignment is heavily penalized. A small penalty is also applied if an assignment is made containing a fucose when no B or Y ions containing a fucose are observed. This, for example, helps decide between a hexose compared to a fucose and an ammonium adduct. The MS-Filter results are displayed using MS-Viewer (16). The default output is filtered to report unique glycopeptides. To report spectral identifications the results were sorted by file-> MSMSInfo (scan number)-> Glyco Score (descending)-> Peptide Score(descending), then replicate identifications of the same file and scan number were removed.

A video tutorial that walks through this analysis process is available at https://vimeo.com/channels/194363/992202630. Protein Prospector results, along with access to annotated spectra, are available through MS-Viewer (16) using search key 9josmlk1s9, or directly accessed through the url: https://msviewer.ucsf.edu/cgi-bin/mssearch.cgi?report_title=MS-Viewer&search_key=9josmlk1s9&search_name=msviewer.

### Glycopeptide Results from Other Software

The glycopeptide identification results used for comparison are previously published results for Byonic, GlycoDecipher and StrucGP (9). New results were created for pGlyco and MSFragger to allow a clearer assessment of the effect of changing one parameter: allowing for ammonium adducts.

pGlyco3 and MSFragger (v21.1) results were created by searching mouse entries from a SwissProt database downloaded on May 25th 2022 (*i.e.* the same protein entries that were searched by Protein Prospector) allowing precursor and fragment tolerances of 5 ppm and 20 ppm, respectively. pGlyco considered the ‘pGlyco-N-Mouse-large’ glycan database (1670 glycan entries) that is one of the default options with both software; MSFragger used the “glyco-N-HCD” workflow and considered the “Mouse-N-glycans-large” set of glycans (identical to those considered by pGlyco). In addition to these glycans, other variable modifications considered by both software included Oxidation[M], Acetyl[ProteinN-term] and Gln-> pyro-Glu[AnyN-termQ]. Results were reported at an FDR of 0.01. The .cfg with full parameters for pGlyco and the .params file for MSFragger are both provided in the supplementary material. For the ammonium adduct search the only parameter changed in pGlyco was including the additional sugar residue ‘aH’, which corresponds to the ammonium adduct of a hexose, and in MSFragger the adduct NH3 was added.

## Results

### Comparison to Previously Published Results

To compare Protein Prospector performance to other software a mouse liver dataset that has been used as part of software comparisons in previous publications (10, 12) was analyzed. This dataset is relatively complicated: it contains N-glycans bearing NeuAc and NeuGc, and a previous publication (10) commented on the presence of spectra with ammonium and iron adducts.

Glycopeptides are more prone to ammonium and metal adducts than unmodified peptides. Protein Prospector can allow for these modifications to expected sugar compositions. Table 1 shows a comparison of the number of spectra identified by different software from this dataset. Two values are reported for Protein Prospector: one is the number of spectra identified when allowing for ammonium and iron adducts to glycans; and a second value when these are not counted.

**Table 1:** Number of spectra identified to glycopeptides in the mouse liver dataset PXD005553 by different software

Software	Spectra: No adducts	Spectra: Including adducts	Unique Peptide + Glycan	Glycopeptide sequences
Protein Prospector	41,787	58,023	6373	1038
MSFragger	27,933		3379	990
GlycoDecipher	27,855		3147	908
Byonic	27,499		3750	1021
pGlyco	23,283		2937	908
StrucGP	17,492		2550	916

Two values are reported for Protein Prospector; one where ammonium and iron adducts were included, and a second when no adducts were counted (which was the case for the searches with the other software). The number of unique glycopeptides and glycosylated peptide sequences are also listed.

Supplemental Table S2 presents a spectrum-level side-by-side comparison of glycopeptide identifications to this dataset. There were 67,174 spectra for which at least one of the six search engines reported a result. There were 222,670 spectra in total in the dataset that contained the m/z 204.087 ion, so a glycopeptide result for 30% of these spectra was reported by at least one software.

**Fig. 1:**
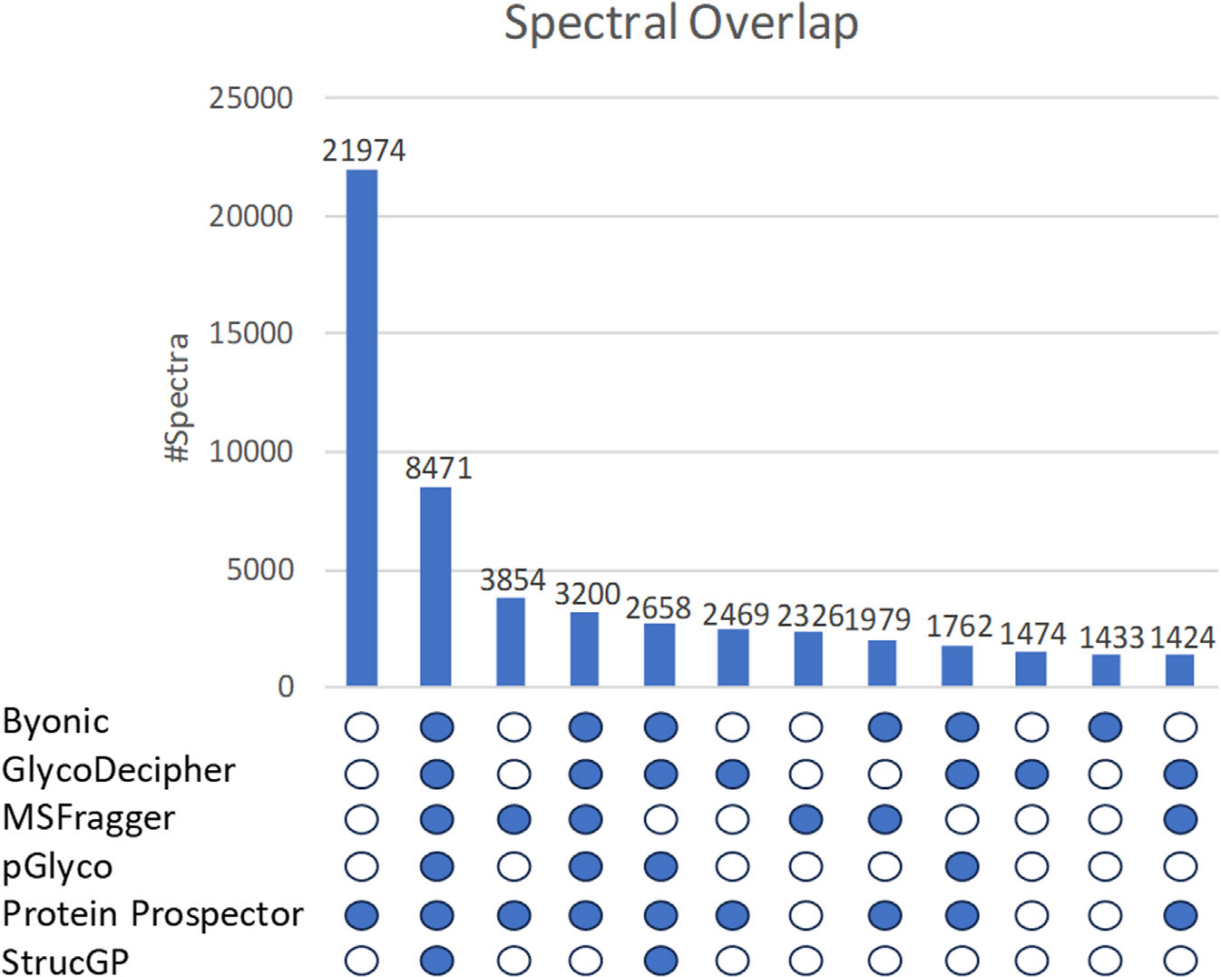
**UpSet plot illustrating the twelve highest overlaps in spectral identifications when six software analyzed a mouse liver glycopeptide dataset (****PXD005553****).** The Protein Prospector results included 16,236 identifications to adduct spectra that were not considered by other software in the compared searches.

Figure 1 shows an UpSet plot of the twelve largest overlaps (or not) in spectral identifications from the software (Supplemental Table S3 lists the complete set of overlaps). There were 8471 spectra for which all six software reported a result. Another 7899 had a result from 5/6 software. At the other end of the scale, 27,998 spectra were only identified by a single software. 21,974 of these were unique to Protein Prospector, partly due to it considering adducts that were not an option in the other searches. This value is reduced when other software consider ammonium adducts (see next section). The largest set of results that were unique to two pieces of software were the 3854 that were reported only by Protein Prospector and MSFragger. As these are the two software that initially just score the peptide identification then score the glycan it is maybe not surprising that they should have significant overlap. There were also 1979 spectra only identified by these two software and Byonic. These three are the software that are less dependent on a good Y ion series for glycopeptide ID. Supplemental Fig. S1 shows an example spectrum identified only by these software. 12 y ions and 5 b ions are observed, giving an extremely confident peptide identification. An extensive series of B ions are also seen, including HexNAcHex1-5 that show the attached glycan is of oligomannose-type. However, the only Y ions are a Y0 2+ and Y1 3+, which is not enough for glycan-first software.

While spectral matches are the initial output from most search engine analyses, unique glycoforms is information that is more meaningful for characterizing the sample. Results from all search engines were collapsed to unique glycopeptides, and for the adduct results from Protein Prospector, the adduct was removed from the assignment, then unique glycopeptides were tabulated and compared. The number of unique glycopeptides reported by each search engine is in Table 1, and Supplemental Table S4 lists each peptide+glycan permutation identified, along with which software reported it. Protein Prospector reported 70% more unique glycopeptides than the next software (Byonic). Considering that Protein Prospector reported 111% more spectral IDs than Byonic, this suggests that several of the extra spectral IDs, particularly the adduct spectra, are of glycoforms that were identified from other spectra, probably in a non-adducted state. If one compares the number of glycosylated peptide sequences reported by the different software (Table 1) the numbers are relatively similar, with Protein Prospector listing 1038 sequences; Byonic 1021 sequences, and all other software a value in the 900s. Hence, the much larger number of unique glycopeptides reported by Protein Prospector are additional glycoforms of peptides mostly reported by other software.

As an example of how unique glycopeptide results compare for a given glycosylated peptide, the alphabetically first glycopeptide identified in this dataset was AACAVRPQEVTMVNGTLTNPVTGK from Cation-independent mannose-6-phosphate receptor. Among software, this peptide was reported with seven different glycans attached. Two of these glycoforms (HexNAc2Hex9 and HexNAc4Hex5NeuGc2) were reported by four different software. The other five were unique to one piece of software: four to Protein Prospector and one to MSFragger. Three of the extra glycoforms reported by Protein Prospector differ by one residue from the glycoforms reported by the four software: HexNAc2Hex8; HexNAc4Hex5NeuAcNeuGc; HexNAc4Hex5NeuGc3. One expects to find closely-related glycoforms, so these are reassuring results. Additionally, the retention times of these extra glycoforms are consistent with their sialylation states: the HexNAc2Hex8 glycoform co-eluted with the HexNAc2Hex9 glycoform around 124 min; the HexNAc4Hex5NeuAcNeuGc version eluted around 151 min along with the other doubly sialylated; and the assigned triply sialylated eluted at 167 min. The other two glycoforms reported are HexNAc4Hex7NeuAc (by Protein Prospector, assigned with an ammonium adduct) and HexNAc4Hex7NeuGc by MSFragger. The correct answer to both is probably HexNAc4Hex5NeuGc2 with an ammonium adduct. These spectra were both acquired around 151 min in the chromatography when other doubly sialylated glycoforms eluted. The spectrum incorrectly reported by Protein Prospector is shown in Supplemental Fig. S2, and contains B ions for both NeuAc and NeuGc, so both the incorrect and correct assignment obtained the same glycan score and it arbitrarily picked (the probably incorrect) one to report. Background contaminating ions in spectra are quantified and discussed later in this manuscript.

Some of the extra identifications are due to Protein Prospector considering a wider range of glycans. The Protein Prospector results identified 249 spectra containing phosphorylated glycans, which none of the default glycan databases available for these other software consider. Supplemental Fig. S3 shows one example of these, containing many glycan fragments containing the phosphate group, including the most frequently observed and diagnostic m/z 243.027 oxonium ion corresponding to Hex+Phospho. There were also 51 glycopeptide spectra in the sample that contain O-acetylated NeuGc. Supplemental Fig. S4 shows an example of one of these spectra, including the diagnostic ions previously reported at m/z 332.098 and 350.108 for the acetylated monosaccharide (17) as well as a peak at m/z 715.240 corresponding to HexNAcHexNeuGcAc from the glycan antenna.

### Adduct Spectral Identifications

Not including adducts, Protein Prospector reported 50% more identifications than the next highest software. Including the adducts Protein Prospector reported 108% more spectral IDs than the next software in these analyses. While searching, three adducts to the glycans were considered: ammonium, iron and aluminum. The number of spectra with each of these adducts is reported in Table 2.

**Table 2:** Number of glycopeptide spectra with different adducts reported by Protein Prospector

Adduct	Spectral IDs
NH3	12,415
Fe[III]	3821
Al[III]	772

So, how reliable are these extra identifications? It is our opinion that there are no aluminum adducts in this dataset; this was considered to allow an estimate of the false discovery rate (these results were removed from all further comparisons). Adducts for a given glycopeptide are nearly always observed at a lower intensity than the fully protonated version. Hence, if the fully protonated equivalent was not also identified this makes any adduct spectral assignment almost certainly wrong, and this was the case for practically all of the aluminum adduct assignments. As there were 772 spectra matched to aluminum adducts, and these were considered to all glycans, one can estimate a similar number of incorrect assignments to non-adducted glycan assignments and each other adduct. This would give an estimated FDR among unmodified peptides of 1.8%, and among the larger 58,023 spectral matches reported by Protein Prospector about 4.0% are estimated to be incorrect.

Supplemental Fig. S5 shows an example of non-adducted, ammonium, calcium (which was not included in the final analysis as there were very few of these) and iron adduct for the same glycopeptide, where the ammonium adduct spectrum looks very similar to the fully protonated, whereas the metal adduct spectra display mass shifts on glycan Y ions, as we have previously reported (14). Considering iron adducts increased the number of spectral IDs by about 9%, whereas ammonium adducts led to 30% more spectral IDs compared to not allowing for adducts.

**Fig. 2:**
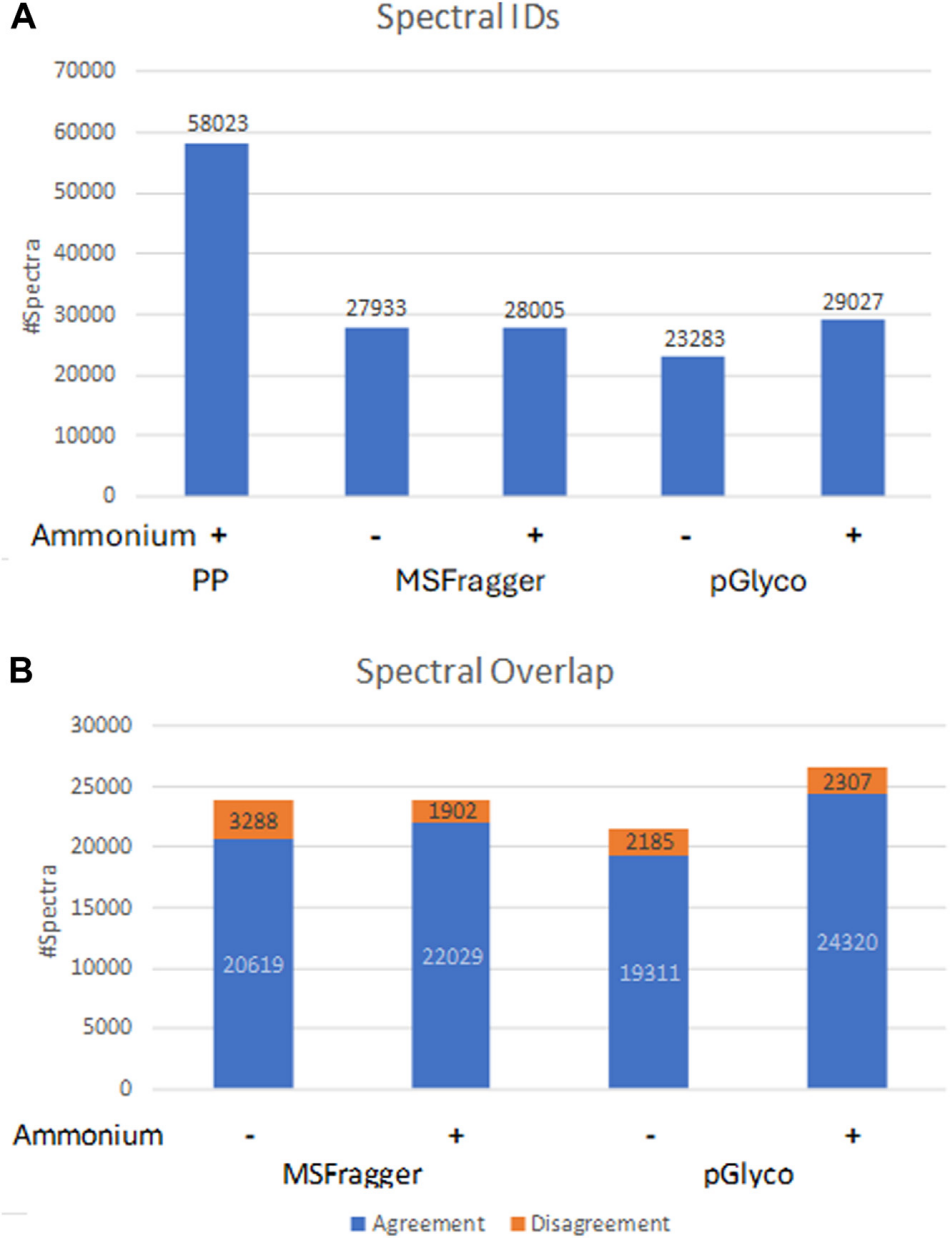
**Spectral Identifications and agreement: the effect of allowing for ammonium adducts.***A*, the number of spectral IDs reported by Protein Prospector when allowing for a range of adducts, and by pGlyco and MSFragger with or without allowing for ammonium adducts. *B*, overlap in spectral identifications between Protein Prospector and pGlyco or MSFragger, and the agreement in the assignment.

Some other software can consider ammonium adducts. Hence, we re-searched this dataset using pGlyco and MSFragger, allowing for ammonium adducts. The change in spectral identification is illustrated in Figure 2*A*. The pGlyco search reported 29,027 spectra; *i.e.* an increase of 5744 spectral IDs, whereas MS-Fragger reported 28,005, only 72 more than the search not considering ammonium adducts. pGlyco reported 6097 results with ammonium adducts; MS-Fragger reported 2568. Hence, pGlyco is reporting a lot of new spectral identifications along with changing some assignments to previously reported spectra, whereas MS-Fragger is almost exclusively changing glycan assignments for spectra it identified with a different glycan in the original search. By allowing for ammonium adducts pGlyco went from reporting 4650 fewer spectra than MSFragger to 1022 more.

Figure 2*B* illustrates the change in overlap of results between Protein Prospector and the searches with these two other software before and after allowing for ammonium adducts. The number of overlapping spectral IDs between Protein Prospector and pGlyco increased from 21,497 to 26,629. Among these the agreement in the assignments went from 19,311 to 24,320, so an increase in 5132 overlapping spectral IDs led to 5009 more assignments that agreed with Protein Prospector. The overlap in spectra with reported IDs between Protein Prospector and MSFragger only went up from 23,908 to 23,932, but the agreement in the assignments went from 20,619 to 22,029; i.e. around 1400 spectra where previously the software had conflicting results now agree.

As MSFragger assigns the peptide first, in each of the 2497 spectra where it has changed its assignment to an ammonium adduct it reported the same peptide as in the other search. Over half of these assignments (1254) were changing a glycan assignment of HexNAc4Hex3NeuAc2 to an ammonium adduct of HexNAc2Hex9 (the latter assignment is 0.994 Da higher in mass). This oligomannose glycan is abundant in the sample, so adducts of this are not surprising. Hence, this new search is correcting a single previously incorrect glycan assignment that alone represented 4.5% of the MSFragger results in the original search. Looking at results reported by other software for these 1254 spectra when not allowing for ammonium adducts, Byonic reported 722 of these with incorrect glycans; GlycoDecipher 6; pGlyco 7; and StrucGP none. Hence, this appears to be a problem mostly suffered by peptide-first searching software.

The comparison of results between the pGlyco searches with and without allowing for ammonium adducts is more layered. As previously stated, it reports 6097 spectra with ammonium adducts. Interestingly, for 130 of these the search not allowing for ammonium adducts reports the same result, but without the adduct; i.e. it essentially got the correct answer even though the precursor mass differed significantly from the reported assignment. There are 475 results where it changed the glycan assignment between the searches. In 30 of these instances this is because it now reports a different peptide for the spectrum. For 410 of these it now reports a NeuGc with an ammonium adduct instead of two hexoses (mass difference of 0.011 Da), most commonly to assign the sugar composition HexNAc4Hex5NeuGc2 (in 277 instances), which corresponds to a fully sialylated biantennary structure; a likely glycan composition. Hence, the search allowing for ammonium adducts is correcting a little under 2% of the glycan assignments by pGlyco.

### Background Glycan Fragment Ions

MS-Filter reports statistics about how often different B and Y ions are observed in spectra, and how often they are present but not expected based on the assigned glycan. The observation of background oxonium ions is common. In glycopeptide-enriched samples the vast majority of spectra contain a m/z 204 ion, even if the main component being fragmented is not a glycopeptide. In this mouse liver dataset 222,670/226,702 (98%) of the spectra contained the ion, but 11,331 spectra containing the m/z 204 ion were identified to unmodified peptides in the Batch-Tag search of this dataset (about a quarter of the spectra identified in this search). The phenomenon of background ions is caused because most glycopeptides produce these ions and some of the oxonium ions give a strong signal, so if there are multiple lower-level glycopeptides co-isolated for fragmentation along with the targeted peptide then they can produce these ghost peaks.

For the purposes of glycan assignment background oxonium ions can mislead software into incorrect glycan assignments, such as in Supplemental Fig. S2. There were 15,198 NeuGc-containing glycopeptide spectra identified by Protein Prospector. However, 40,466 glycopeptide spectra identified by Protein Prospector as not containing a NeuGc contained the m/z 290 oxonium ions and 34,149 contained the m/z 308 oxonium ion at some intensity; i.e. practically every identified spectrum contained a NeuGc oxonium ion, but 70% of the identified glycopeptides did not contain the monosaccharide. A full list of background B ions observed is given in Supplemental Table S5. Glycan rearrangement is another phenomenon that can lead to unreliable glycan structural assignments. This is a phenomenon best documented for fucose (18). However, it does occur for other residues. Of the glycopeptide spectra identified by Protein Prospector 13,708 (23%) contain a Y ion peak corresponding to the peptide + HexNAcHex. Supplemental Fig. S6 shows an example spectrum with the Y+HexNAcHex peak highlighted. This is a fragment that cannot be produced from an N-linked structure without a rearrangement. These peaks were nearly always less than 5% of the intensity of the Y1 ion, but rearrangements are something that must be borne in mind if peak intensity is not considered.

## Discussion

We present Protein Prospector’s innovative way to identify more glycopeptide spectra. The process follows three steps. It starts by filtering for probable glycopeptide spectra, usually based on the presence of the HexNAc oxonium ion. It then reports confident glycopeptide identifications based on database searching. Finally, it identifies additional glycoforms of these peptides based on the presence of Y0/Y1 ions and performs glycan scoring to determine the best matching glycan based on the data. This approach works for both N- and O-glycosylation, although the benefit is expected to be more significant for N-linked as there are typically more glycoforms for a given modification site. It works best when analyzing collisional fragmentation data, where Y0 and/or Y1 ions are reliably observed. We have also evaluated its use for EThcD data (not shown) where for N-linked glycopeptides its benefit is less consistent, as at typical supplementary collision energies Y1 ions may not always be seen, so it helps for some glycopeptides, but not all. It should be highlighted that these steps can also be performed in isolation and combined with other software tools. For example, a peak list file filtered for presence of oxonium ions could be used as an input to an alternative glycopeptide analysis software. Similarly, one could take a list of peptides identified by alternative software as glycosylated and input this into MS-Filter to try to identify additional glycoforms and re-assess glycan assignments.

In this study, we have demonstrated that Protein Prospector can identify more glycopeptide spectra than competing tools. It should be noted that the estimate for the FDR among the Protein Prospector results is slightly higher than the other software’s estimates for their data, although the accuracy of any of these estimates is questionable. It achieves this partly because it routinely considers a wider range of glycans (although the number of glycans it considered was actually less than half that considered by MSFragger and pGlyco (730 vs 1671), and also because it can consider several glycan modifications or adducts. While the adducts can have a significant impact on the number of glycopeptide spectra that are identified, Protein Prospector reported many more results than other software even when these are not considered. Examples of less-considered glycoforms mentioned in this article include glycans containing mannose-6-phosphate and O-acetylated NeuGc. The former of these can be considered by some other software used in this comparison, although one would have to manually add these glycans; the latter we believe is not an option in any of these other tools. The finding that allowing a large glycan search space is beneficial for glycopeptide identification was one of the observations of the community study comparing glycopeptide software published a few years ago (6), and the current results support that conclusion.

While Protein Prospector reported more than twice as many spectral IDs as other software, the increase in unique glycopeptide IDs was smaller, and the number of glycosylated peptide sequences reported by Protein Prospector was only marginally higher than other software. This highlights that the novel step in the Protein Prospector workflow making use of Y1 ions is detecting additional glycoforms of peptides mostly found by other software: it is reporting on average about six glycoforms per peptide identified, whereas other software report 3 to 4 per peptide. The frequency of reporting a certain number of glycoforms for an identified glycopeptide is plotted in Supplemental Fig. S7. For all software other than Protein Prospector glycopeptides with a single glycoform are most commonly reported, with a gradual tailing in frequency of higher number of glycoforms. However, Protein Prospector reports glycopeptides with 1 to 4 glycoforms all at similar frequency before tailing away for higher numbers of glycoforms.

Software that performs glycopeptide identification at the omic level is often described as being either peptide-first or glycan-first. The three software that produced the most unique glycopeptide IDs were Protein Prospector, MSFragger, and Byonic. The first two of these perform peptide identification and then try to determine the attached glycan, whereas Byonic tries to assess peptide and glycan ID in one step, so these represent software that is trying to identify the peptide in the initial step. However, it would be wrong to conclude that peptide-first analysis is more sensitive. The glycan-first approach can identify glycopeptides where there is inferior peptide fragmentation, as it only considers peptides containing an N-glycosylation motif for spectra it has decided is of an N-glycopeptide, whereas peptide-first will additionally consider these spectra as being of non-glycosylated peptides, so needs more peptide fragments to reach a given FDR threshold. The new strategy Protein Prospector is employing compensates to some extent for this, but it still requires at least one confident identification of a given glycopeptide sequence before other glycoforms can be matched in MS-Filter. Where the peptide-first search engines outperform the glycan-first is when the glycan fragment ions are fewer; for example, if the glycopeptide is heavily fragmented such that only the Y1 ion and one or two other Y ions remain (see Supplemental Fig. S1 for an example), or if the N-glycan is truncated, so there are few Y ions possible. In these situations, the peptide-first software assigns the glycan mostly based on mass, although there may be many supporting B ions. For smaller glycans, this is still fairly reliable, but for larger glycans we show in this study that this software is likely to make more incorrect glycan assignments than glycan-first if the correct answer is not considered, particularly for spectra of N-glycopeptides with ammonium adducts.

We also highlight the strengths and weaknesses of allowing for adducts. In the case of Protein Prospector (and probably also other search engines) allowing for an adduct may double the number of incorrect glycopeptide identifications, as it is doubling the glycan search space. For an adduct that is not common in the dataset considering this may lead to an increase in the incorrect identifications while gaining little or no benefit. In our initial analysis of this data set we also considered sodium and calcium adducts. There are some calcium adduct spectra in this dataset (one is shown in Supplemental Fig. S5). However, the number of correct calcium adduct spectra is dwarfed by incorrect assignments, so including these results would have made overall results less reliable for little real gain. However, some adducts can be common, and considering these not only leads to more total identifications but will also rectify results that would be incorrectly assigned if the adduct was not considered. The occurrence of adducts is quite variable depending on the dataset, but they are not unusual (certainly dramatically more common than in datasets of unmodified peptides). Ammonium adducts seem to be by far the most common in glycopeptide datasets, and they are the adduct that is most likely to lead to incorrect glycan assignments. In this study we focused on a previously produced mouse liver dataset (12). Data from four other tissues were produced at the same time, and based on previous analysis using GlycoDecipher of all these tissue data (9), all datasets contained some ammonium adducts, but the liver dataset contained the most, and was one of two that contained iron adducts.

We believe Protein Prospector is currently unique among glycopeptide software in being able to consider adducts on glycan Y ion fragments. These are rarely seen in ammonium adduct spectra but are common for metal adducts. These shifted ions could cause problems for other software as they cannot annotate them. They could also lead to glycan-first software misinterpreting the mass of the peptide as including the mass of the adduct, meaning they will not identify the spectrum. However, in this study we did not evaluate the performance of other software in identifying metal adduct spectra. The searching strategy presented here for Protein Prospector did not consider adducts in the database searching using Batch-Tag; these were only added in the subsequent MS-Filter analysis. The MS-Filter search is very quick (a few minutes for this dataset), so it is quite practical to initially allow for many types of adducts, look at the results, then reduce those considered when repeating the MS-Filter step to only those that appear to be present (remembering that there will be random matches to all adducts, so evaluation of whether they are real should be based on whether the same peptide glycoforms reported with a given adduct are also observed without the adduct).

Most software tools are continually developed. The latest versions of pGlyco3 and MSFragger were used in this study. The other results were those acquired in 2022. According to their relevant github pages there has not been a new release of StrucGP since then, whereas GlycoDecipher has progressed from v1.0.2 (probably) to v1.0.4, so there may have been some minor improvements in the interim. We have no information about changes in Byonic between now and then. The way the raw data was acquired can also affect how well given software performs. For example, among the software compared here StrucGP has been optimized for data acquired with specific stepped HCD collision energies that differ slightly from those used to acquire this dataset, so may have performed better on data acquired slightly differently. The other tools have been used on a wider variety of data, but raw data could still be a variable in the performance.

Previous comparisons of proteomic and glycoproteomic software have highlighted that the user (specifically their expertise with a particular piece of software) is at least as significant a variable in performance as the software itself (6). One might assume that as developers of Protein Prospector, we have used relatively optimal parameters for the results we present for our software. The GlycoDecipher results are also those produced by developers of that software, so should represent top performance. However, the results for the other four tools were produced by users who are more familiar with other software, so probably represents more average performance for users of these software.

The searches with and without adducts also highlight the inaccuracy of glycan FDR estimates reported by software, where in the case of MS-Fragger nearly 5% of the identifications should have been to an ammonium adduct of a single sugar composition that was not in the search space of this search. These were all incorrectly assigned at a supposed 1% glycan FDR, and this is only a subset of the IDs that were presumably wrong in the search that did not consider ammonium adducts. It seems that for peptide-first glycopeptide analysis software such as Protein Prospector, MS-Fragger, and Byonic, it is particularly important to have a search space that includes as many correct answers as possible, as these are more likely to identify the correct peptides but with the wrong glycan composition, whereas in these situations the glycan-first software is more likely to not report anything, although even these make errors higher than their estimated FDR if the correct answer is not a possibility.

Part of the reason why software is inaccurate in its FDR estimation is that there are instances when the glycan assignment is ambiguous based on the data, especially if one allows for incorrect monoisotopic peak assignment and ammonium adducts, and in this situation all software currently pick one assignment. In this manuscript we highlighted an instance where Protein Prospector probably made an incorrect assignment containing NeuAc, when the correct answer contained NeuGc. Unique ions supporting the presence of both sialic acids were present in the spectrum (see Supplemental Fig. S2); the only way to differentiate between them would be based on relative peak intensity. This is an example where background ions have compromised the ability of software to get a confident result. In some cases, a very tight mass tolerance can remove ambiguity, but in our experience, there are normally some correct identifications with outlier mass accuracies; maybe because some glycopeptide precursors have weak monoisotopic peaks due to their high mass and low intensity. It is worth highlighting that the ability of Protein Prospector to provide annotated spectra and to compare different glycopeptide assignments to the same spectrum are key advantages over other glycopeptide software when trying to troubleshoot such issues.

Protein Prospector is currently not reporting glycan topology; only composition, whereas some other software does. The MS-Viewer output of the MS-Filter results contains columns for all potential B and Y ions and the intensity of the peak, if it was observed. Hence, the information to deduce some topology is present in the output, and in future versions will be made use of for higher specificity of the glycan assignment.

Analysis of glycopeptide spectra is not easy. While several pieces of software have been produced or improved since the community study published in 2021 (6), all software still has plenty of room for improvement. In this manuscript we have provided an updated performance measure for Protein Prospector that shows it seems to still outperform newer leading software, although we highlight that reliability estimates of all these software may not be particularly accurate. We also demonstrate that glycan search space is a key parameter in both the number and reliability of glycopeptide identifications.

## Data Availability

Protein Prospector is freely accessible for use as a web-based search engine at prospector.ucsf.edu. It can also be downloaded for installation locally upon request.

A video tutorial that walks through the Protein Prospector analysis process presented in this manuscript is available at https://vimeo.com/channels/194363/992202630.

Protein Prospector results in this manuscript, along with access to annotated spectra, are available through MS-Viewer (16) using search key 9josmlk1s9, or directly accessed through the url: https://msviewer.ucsf.edu/cgi-bin/mssearch.cgi?report_title=MS-Viewer&search_key= 9josmlk1s9&search_name = msviewer.

## Supplemental data

This article contains supplemental data.

## Conflict of interests

The authors declare the following financial interests/personal relationships which may be considered as potential competing interests: R. J. C. and P. R. B. are Editorial Board Members of *Molecular and Cellular Proteomics*, but were not involved in the editorial review or the decision to publish this article.

## Supplementary Data

Supplemental Tables

Glycan_Scoring

Fragger.params

pGlyco.cfg
