# Supplementary material for: Improving the Depth and Reliability of Glycopeptide Identification Using Protein Prospector
Source: Mol Cell Proteomics. 2025 Jan 7;24(2):100903. doi: 10.1016/j.mcpro.2025.100903 (PMC11851224; doi:10.1016/j.mcpro.2025.100903)
Supplement: Supplementary Figures [file mmc1.docx]

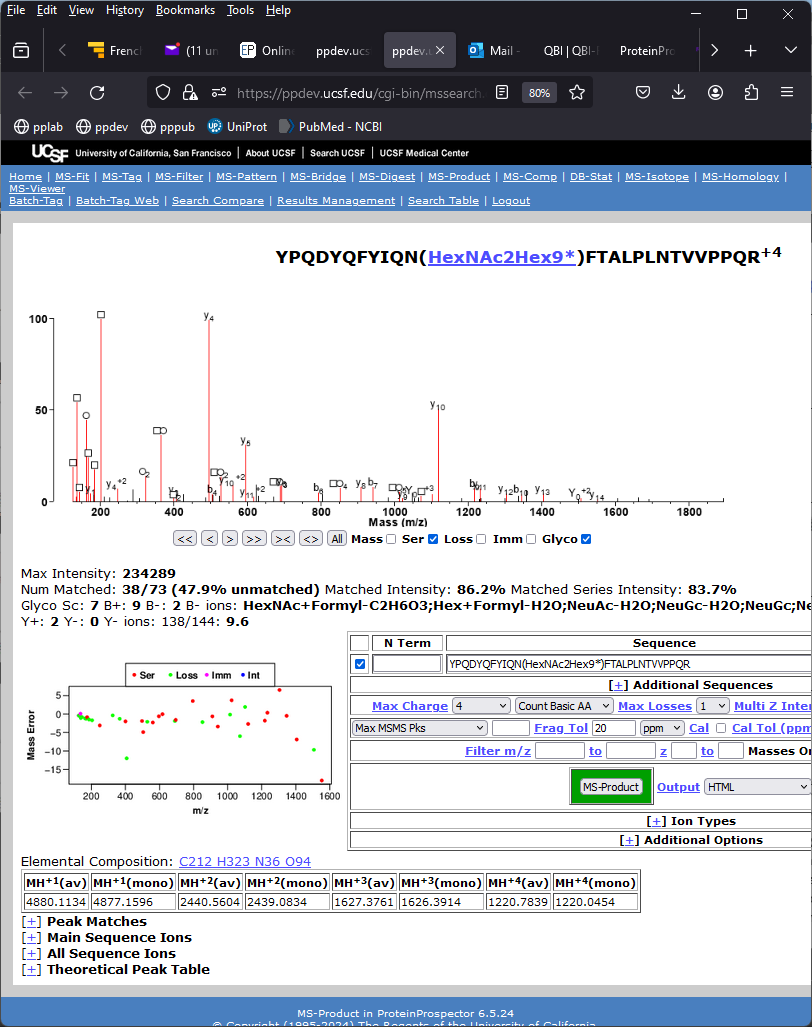


*Supplementary Figure 1: Example of a spectrum identified by search engines Byonic, MSFragger and Protein Prospector, but not by glycan-first tools. The glycopeptide YPQDYQFYIQNFTALPLNTVVPPQR is found in Translocon-associated protein subunit alpha. This is scan 53586 in raw file ‘MouseLiver-Z-T-1’. The spectrum only contains two Y ions: a Y0 2+ and Y1 3+.*


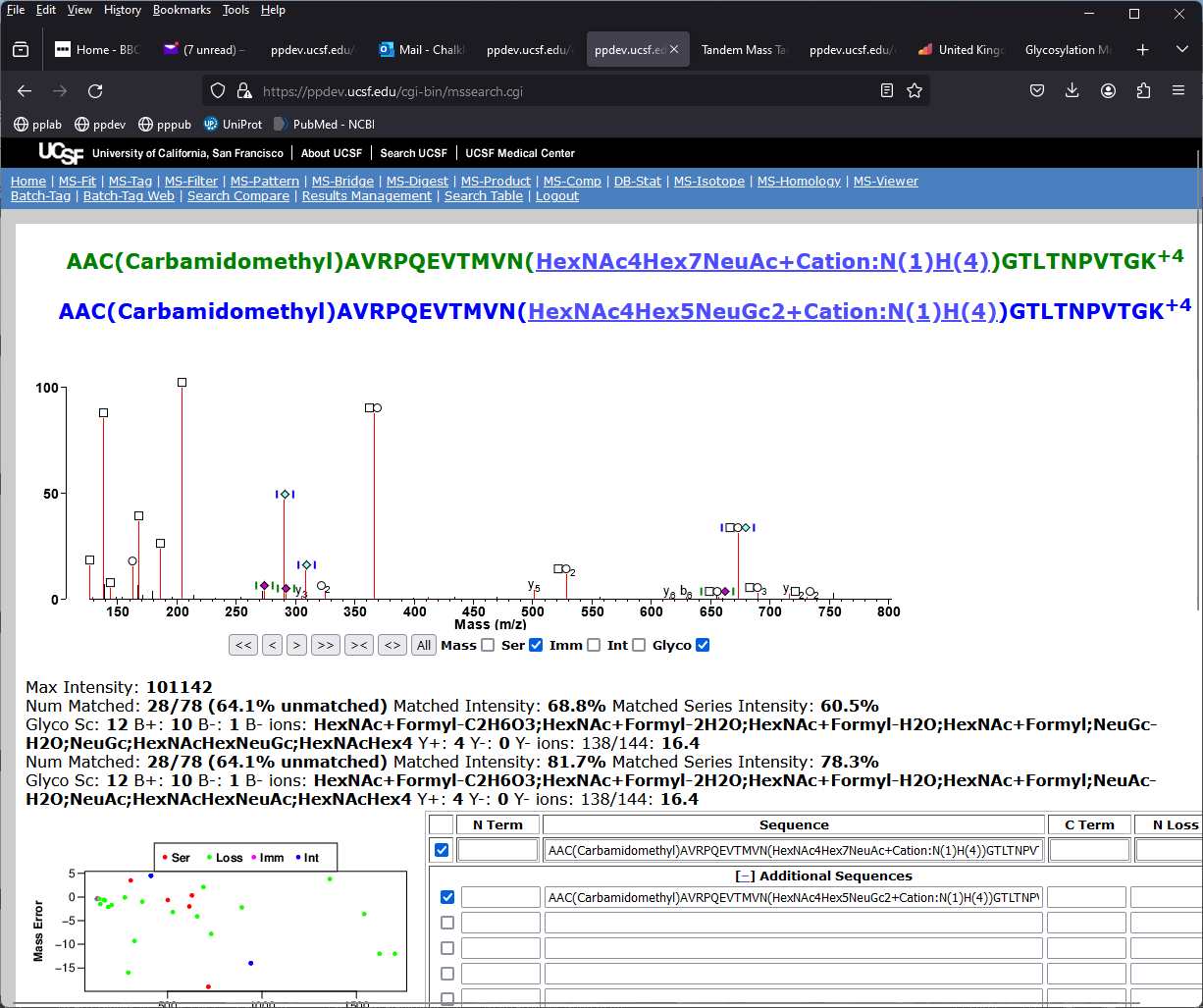


*Supplementary Figure 2: Comparison of two potential glycan assignments to the same spectrum. Scan 32769 from fraction MouseLiver-Z-T-2 was assigned by Protein Prospector to a peptide from Cation-independent mannose-6-phosphate receptor with the glycan HexNAc4Hex7NeuAc with an ammonium adduct, whereas the correct answer is probably HexNAc4Hex5NeuGc2 with an ammonium adduct. This figure shows the lower mass half of the spectrum and highlights that ions supporting the presence of both NeuAc and NeuGc are observed; albeit the NeuAc ions are of lower intensity. Annotated glycan fragment Ions surrounded by green or blue \ are only consistent with the green or blue peptide assignment. The NeuAc ions probably should not be there: they are likely background contamination in the spectrum.*

*Supplementary Figure 3. Annotated spectra of a glycopeptide NTTSAAMVHCLR from Carboxylesterase 1E. The glycan, attached to Asn76 of the protein, contains a mannose-6-phosphate with the outer GlcNAc still attached. This spectrum is scan 14710 in raw file ‘MouseLiver-Z-T-1’. (a) Annotated full spectrum showing peptide and glycan fragments. (b) Plot of only glycan fragments, where ions to the right of ‘0’ correspond to glycan oxonium ions, whereas ions to the left of ‘0’ correspond to additional mass of Y ions compared to the unmodified peptide. An essentially complete Y ion series is observed to confirm the third HexNAc is probably attached to the phosphate. Glycan annotations include a square for HexNAc; circle for Hex and p for phosphate.*

*Supplementary Figure 4: Annotated spectra of a glycopeptide FTCNQTTDVIIIHSK from aminopeptidase N. The glycan, attached to Asn114 of the protein, is modified by a biantennary glycan containing a terminal NeuGc on one arm and NeuGcAc on the other arm. This spectrum is scan 28179 in raw file ‘MouseLiver-Z-T-1’. (a) Annotated full spectrum showing peptide and glycan fragments. (b) Plot of only glycan fragments, where ions to the right of ‘0’ correspond to glycan oxonium ions, whereas ions to the left of ‘0’ correspond to additional mass of Y ions compared to the unmodified peptide.*

*Supplementary Figure 5: HCD fragmentation spectra of the glycopeptide DNATDSVPLR from Prolow-density lipoprotein receptor-related protein 1 with a high mannose HexNAc2Hex8 glycan attached to Asn2128. (a) Fully protonated; (b) with an ammonium adduct; (c) with a calcium adduct; (d) with an iron adduct. Note: the ammonium adduct does not affect the fragmentation spectrum as no fragments retain the modification, whereas for the two metal adducts many of the Y ions are shifted by the mass of the metal. These spectra are all from the raw file ‘MouseLiver-Z-T-2’ and are scans 11752, 11763, 11756 and 11817 respectively.*

*Supplementary Figure 6: Example spectrum containing Peptide+HexNAcHex ion that can only be formed by a rearrangement/migration event. This HCD fragmentation spectrum of N-linked glycopeptide DKNGTR from Hypoxia up-regulated protein 1 is scan 296 from fraction MouseLiver-Z-T-1. The Y0+HexNAcHex peak (highlighted by a blue box) is about 3% of the intensity of the most intense fragment peak Y0+HexNAc, and more intense than some glycan fragments that are annotated and expected.*

Supplementary Figure 7: Histogram plots of number of glycoforms identified per glycopeptide by each software.
