# Supplementary material for: Improving the Depth and Reliability of Glycopeptide Identification Using Protein Prospector
Source: Mol Cell Proteomics. 2025 Jan 7;24(2):100903. doi: 10.1016/j.mcpro.2025.100903 (PMC11851224; doi:10.1016/j.mcpro.2025.100903)
Supplement: Glycan_Scoring [file mmc3.docx]

Glycan Scoring in Protein Prospector

Glycan scores in Protein Prospector are calculated by summing the number of B and Y ions observed that are consistent with the reported glycan assignment, then subtracting points for every glycan fragment observed that is inconsistent with the assignment. No intensity threshold is applied. Observed glycan ions that are consistent with a glycan assignment score +1; those inconsistent score -2.

There are some additional adjustments to this scoring:

- The fragments of the HexNAc oxonium ion (m/z 126.055, 138.055, 144.066, 168.066, 186.076) do not count to the glycan score, as these do not differentiate between different glycan assignments.
- Certain B ions are so commonly seen as background in spectra that their presence are not counted against the glycan score. These ions include:
  - NeuAc-H2O 274.092
  - NeuAc 292.103
  - HexNAcHexNeuAc 657.235
  - NeuGc-H2O 290.087
  - NeuGc 308.098
  - HexNAcHexNeuGc 673.230
- Assignments of glycans containing NeuAc, NeuAcAc NeuGc, NeuGcAc or phosphate are penalized ten points if no glycan fragment ions containing the relevant sugar or phosphate are observed, as these are essentially always observed for these types of sugars.
- Assignments containing fucose are penalized two points if no glycan fragments containing a fucose are observed. This functions essentially as a tie-breaker when deciding between assignments containing an extra hexose versus one with a fucose and an ammonium adduct (with incorrect monoisotopic peak assignment)
